# Commercial Video Games As Therapy: A New Research Agenda to Unlock the Potential of a Global Pastime

**DOI:** 10.3389/fpsyt.2017.00300

**Published:** 2018-01-22

**Authors:** Michelle Colder Carras, Antonius J. Van Rooij, Donna Spruijt-Metz, Joseph Kvedar, Mark D. Griffiths, Yorghos Carabas, Alain Labrique

**Affiliations:** ^1^Johns Hopkins Bloomberg School of Public Health, Baltimore, MD, United States; ^2^Johns Hopkins University Global mHealth Initiative, Baltimore, MD, United States; ^3^Trimbos Institute, Netherlands Institute of Mental Health and Addiction, Utrecht, Netherlands; ^4^Dornsife Center for Economic and Social Research, University of Southern California, Los Angeles, CA, United States; ^5^Harvard Medical School, Boston, MA, United States; ^6^Nottingham Trent University, Nottingham, United Kingdom

**Keywords:** video games, mental health, prevention, technology, social media, eHealth, Internet, social support

## Abstract

Emerging research suggests that commercial, off-the-shelf video games have potential applications in preventive and therapeutic medicine. Despite these promising findings, systematic efforts to characterize and better understand this potential have not been undertaken. Serious academic study of the therapeutic potential of commercial video games faces several challenges, including a lack of standard terminology, rapidly changing technology, societal attitudes toward video games, and understanding and accounting for complex interactions between individual, social, and cultural health determinants. As a vehicle to launch a new interdisciplinary research agenda, the present paper provides background information on the use of commercial video games for the prevention, treatment, and rehabilitation of mental and other health conditions, and discusses ongoing grassroots efforts by online communities to use video games for healing and recovery.

## Introduction

In 2017, over 130 million Americans (40% of the population) played commercial video games (also known as digital games) (1). Substantial research attention around this now-mainstream habit has examined problems related to video gaming ranging from sedentary screen time, exposure to violence, and excessive or problematic gaming (2, 3). However, there is emerging empirical research into the area of commercial video games as therapy (VGTx). In contrast to the custom-made, video game-based health interventions and applications (games for health) developed by a large community of innovators in mHealth and eHealth, the present paper focuses on commercial, off-the-shelf video games (COTS games) that are designed for entertainment, with no consideration of their therapeutic potential. In a sense, one might consider VGTx as a different take on games for health. Instead of making a game as a way to address a health problem, we suggest adapting or incorporating games that millions of people already play into interventions that promote health.

Key aspects of COTS games and their associated technologies—from the virtual gameplay-centered social support networks to the highly engaging, realistic interactive environments—may disrupt healthcare over the coming decade. The level of technological and gameplay sophistication and subsequent user experience made possible through massive corporate funding for COTS games are often orders of magnitude superior to the budgets available to develop bespoke games for health. [It may come as a surprise to many that for some years now, the video game industry’s annual sales (~US$24billion) have been more than double Hollywood movie box office sales (~US$11billion) (4, 5)]. We position this research and action agenda as a call for investigators, mental health professionals, the video game industry, and the gaming community to work together to better understand the opportunities and challenges that this emerging field of innovation presents.

The small body of research in this sphere to date has focused on the impact of unmodified commercial games as health interventions. Pilot studies of games and gaming communities have begun to provide early evidence of outcomes in a variety of health areas [Table 1; for a recent review, see Griffiths et al. (6)].

**Table 1:** Selected studies of video games and health outcomes.

VGTx health function	Condition or population	Game/system identified	Reference
Assessment and monitoring	Physical activity	Pokémon GO	Althoff et al. (7)
	Cognitive status in elderly	FreeCell	Jimison et al. (8)

Cognitive distraction	Anxiety, nausea in chemotherapy	Participant’s choice of 25 games	Redd et al. (9)
	Preoperative anxiety	Choice of 10 games on hand-held system	Patel et al. (10)
	Fibromyalgia pain	Sports games played with Nintendo Wii, Playstation 3, and Microsoft Kinect	Mortensen et al. (11)

Mental health	Depression	Bejeweled 2, Peggle, Bookworm Adventures	Russoniello et al. (12)
	Posttraumatic stress disorder	“Realistic military-themed FPS games”	Elliott et al. (13)
	Improvement of positive symptoms in schizophrenia	Internet games (gambling, role-playing, strategy, shooter)	Han et al. (14)

Neurological rehabilitation	Attention deficit disorder	Car racing games, skateboarding games, or adventure games	Pope and Palsson (15),
	Minimal brain damage, attention problems	Super Breakout	Larose et al. (16)
	Stroke	Wii Sports, PlayStation EyeToy games	Yong Joo et al. (17)

Prevention	Intrusive memories from trauma	Tetris	Iyadurai et al. (18)

Psychotherapy	Assessment of clinical presentation	Various, including Lego Star Wars II	Ceranoglu (19)
	Rapport and treatment	Super Mario Bros., Jeopardy, (The Legend of) Zelda	Gardner (20)

Social skills training	Autism	Pacman	Gaylord-Ross et al. (21)
	Autism	Guitar Hero	Blum-Dimaya et al. (22)

For example, success in playing the computer solitaire game FreeCell may be useful to monitor cognitive status in adults with mild cognitive impairment (8), while new augmented reality games such as Pokémon GO could be useful to promote physical activity among those who are normally reluctant to engage (7). For children undergoing surgery, a hand-held game was more useful in relieving preoperative anxiety than a dose of midazolam (10). Puzzle games such as Tetris and Bejeweled have been shown to reduce depression, stress, and even prevent flashbacks after a traumatic event (12, 18, 23). The structural characteristics of games may provide unique affordances that traditional therapies do not offer.

Games may also be a useful tool in psychotherapy for assessment, building rapport, and providing social skills training (19, 22). Family members or loved ones may also play a useful role in VGTx. For example, playing non-competitive and non-violent video games together is a good way for parents to participate in a child-directed activity in Parent-Child Interaction Therapy [(24) p. 207]. Playing together as a family also fosters social connection between grandparents and grandchildren, which is highly supportive of the health of older adults (25).

As gamers are able to join forces to defeat common virtual enemies or accomplish virtual tasks, gaming communities also unite around real-world problems in a therapeutic and philanthropic way. Gamers and gaming-related organizations—directly and indirectly through charitable contributions—provide social and psychological support, including peer support, online clinician-delivered services, information about mental health conditions, and assistance finding in-person mental health treatment to community members and the gaming population at large. For example, non-profit organizations, such as Stack-Up and Anxiety Gaming, provide spaces where gamers can learn about mental health problems, seek support and assistance, and interact socially either in person or online. This image of connected, socially engaged gamers challenges the stereotypical notion of video game play as an isolating and individual pastime that reinforces societal disconnectedness (26). Growing evidence suggests that online communities are, for specific types of players, socially liberating and contribute to improvements in self-esteem and control of emotions in real-world settings (27). Although it may seem counterintuitive to suggest that individuals with social anxiety or other reasons for avoiding face-to-face social interactions may benefit from interventions that do not involve exposure to “*in vivo*” therapy, online interactions allow individuals with mental health challenges to receive much needed social support and a sense of connectedness or belonging (28, 29), which are ideal interventions for individuals with suicidal ideation and behavior (30).

## Improving Methodological Practice

The serious academic study of VGTx faces several challenges. First, evidence synthesis around VGTx is difficult. There is no standard terminology for commercial video games or gameplay in the medical and psychological literature, and even studies about “serious video games” (i.e., games developed specifically for therapeutic purposes), do not always use that term, instead using terms, such as “interactive digital rehabilitation technology” or simply “virtual reality” (31, 32). Some of the difficulty in defining and naming terms may also be due to researchers’ extant biases and attitudes toward commercial games. Published studies concerning video games from public health, pediatrics, psychiatry, and psychology perspectives appear to have become less positive over time, and studies having a positive focus are more likely to be found in journals with a low impact factor (33). Reviews of the therapeutic effects of video games usually conflate commercial games and custom-designed games or gamified interventions, making it difficult to compare interventions and draw conclusions about the potential benefits of popular commercial video games.

**Figure 1:**
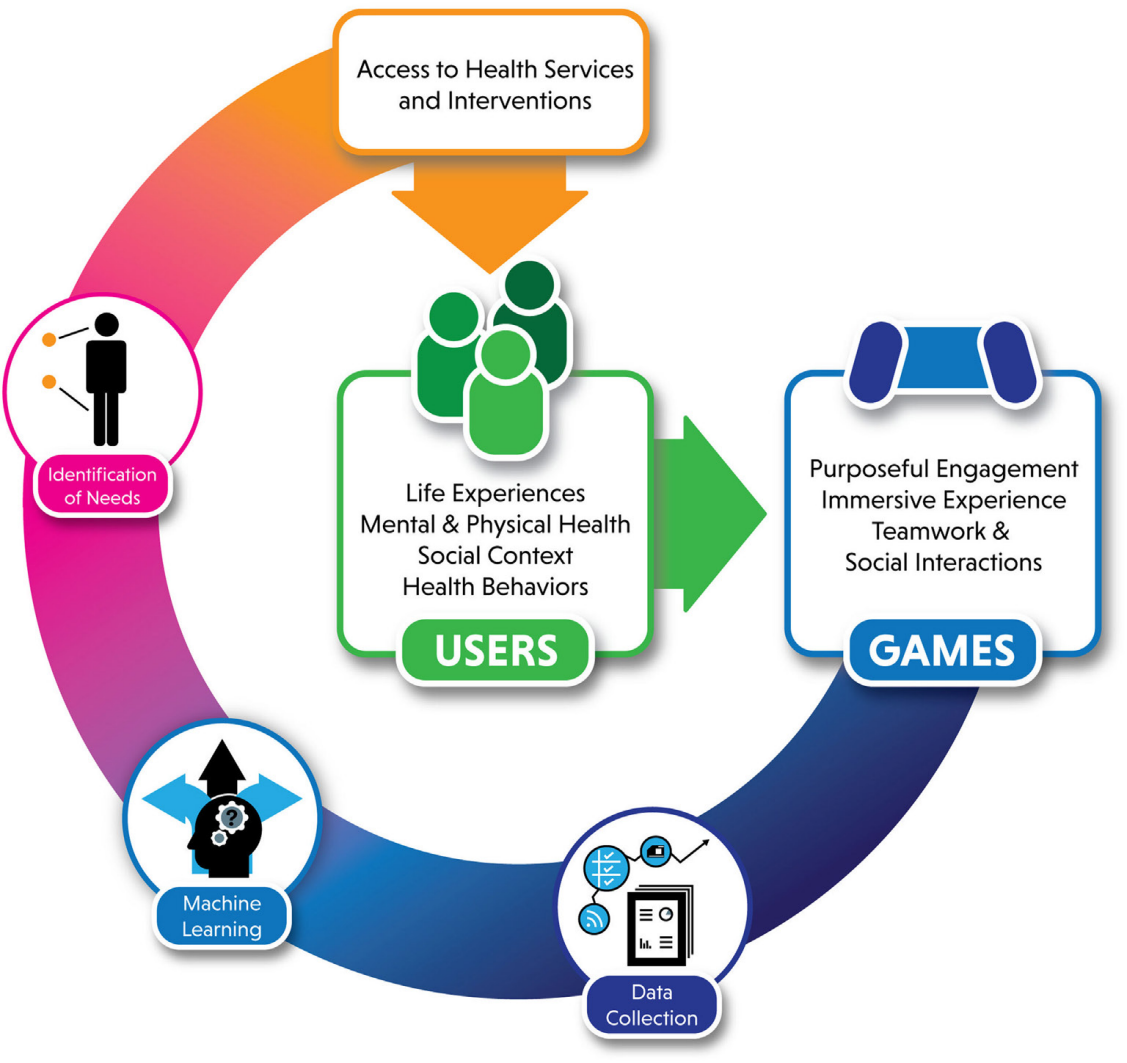
Conceptual framework for video games as therapy.

Second, rapidly changing technology requires a prepared and adaptive health research ecosystem. The pace of growth in technology related to mHealth, eHealth, and games for health research over the past decade has greatly increased and similar changes in video games themselves challenge the ability of public health research to keep up. COTS games would be considered complex interventions, making them among the most challenging to develop and evaluate. In addition, the emergent nature of gameplay experiences adds additional complexity to understanding the interactions between the traditional individual, social, and cultural determinants of health. As described in Figure 1, the individual and social context of users may drive video game play, which offers specific benefits such as purposeful engagement and social interactions that could also form the basis of interventions.

Gameplay itself may provide a data source that could potentially be used to identify needs or opportunities for specific interventions that could then be delivered to users, such as suicide prevention information or in-game peer support. For example, passive data capture of time spent playing games or in-game social interactions and activities (e.g., cooperative play, sending in-game messages) might be an indication of symptom strength in disorders such as schizophrenia or depression (34). Machine learning algorithms that use the “big data” resulting from video game play (Figure 1) may one day be a way to screen players for mental health states and offer intervention the way Twitter feeds have been used to predict depression, bipolar disorder, and PTSD (35). Because of this complexity, creating a science of VGTx involves many of the same challenges as recent efforts to bolster the science of digital health. Technology outpaces research, requiring an interdisciplinary approach that combines methodological rigor with rapid evaluation and changes in research capacity and infrastructure (36).

Games and gaming communities arguably represent complex populations and systems, the study of which requires interdisciplinary collaboration. For example, clinical and public health research may focus on the mainstays of health determinants, such as dose, delivery, and sociocultural contexts, but structural characteristics unique to games also need to be considered—ranging from social and immersive features and reward structures to the controllability of game experiences that contribute to beneficial (and problematic) outcomes (37). For example, it may be the high visuospatial task demand of Tetris that serves to disrupt memory consolidation after trauma, and since adherence to traditional exposure-based therapies for PTSD is low (38), this form of cognitive “vaccine” may be a particularly useful intervention. These structural characteristics are usually the purview of games studies researchers in the fields of communications, ergonomics, media psychology, and health psychology. In these latter fields, a substantive literature on game studies may be found, a body of work that is seldom consulted in the course of public health or medical research. Taking a broader perspective that involves attention to the complex and dynamic nature of gaming and incorporates rigorous scientific methods from various fields to study gaming effects and in-game events is critical to better understanding the individual and population health outcomes from therapeutic uses of games and game communities.

**Figure 2:**
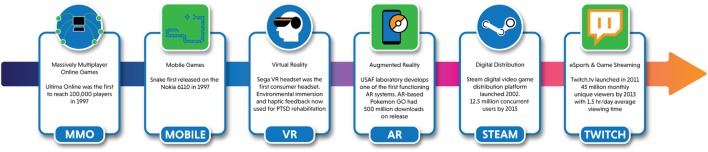
Important milestones in the evolution of video games and gaming communities.

As with any technology-based intervention, public health research must find ways to keep pace with rapidly changing games and game-related technologies. Gaming communities and experiences evolve rapidly, but health research is notoriously slow (39). In the last 40 years, the video game space has moved from static, disconnected games such as Pong and Pac-Man to Internet-based multiplayer games to entertainment portals like Twitch (Figure 2). Twitch, a website and video streaming service devoted to video game play, amassed a following of 45 million viewers in its first 2 years (40). Game “streamers” broadcast and narrate their gaming and interact with viewers verbally while viewers post messages to streamers and other viewers in chatrooms. These 45 million gamers comprise a new type of population that is inherently reachable due to their use of an online gaming-community-based platform. The fast pace of change in gaming communities, technologies, and game features, driven by the demands of game designers (and the players they design for), is not friendly to the years or decades it may take to fund, study, and implement medical and public health innovations (41, 42). While health researchers may take months to over a year to write a grant application and find funding, a new video game community may be formed and grow to millions of individuals or a groundbreaking technology may become commercially available, making an older one obsolete.

Second, software and hardware developers often alter game mechanics or features that change fundamental aspects of a game to improve or adjust the user experience, thus influencing any game-specific research activity and subsequent conclusions. For example, years of game studies research have discussed the social benefits of belonging to a “guild,” “clan,” or other team of players in massively multiplayer online games such as World of Warcraft (43–46), but guild membership is no longer necessary to achieve high-level game accomplishments since gameplay changes were implemented in 2011 and 2013 (47). This is an important difference from the study of custom-made games for health because existing game features and changes to commercial games are market driven by industry developers. Researchers and developers of VGTx may, therefore, have to be able to rapidly adapt or concentrate on therapeutic interventions/assessments that are not dependent on specific game features (e.g., developing interventions based on a type of computer game such as Solitaire) (8, 48) rather than focusing interventions on more specific features such as guild membership. The aforementioned challenges may be addressed by developing constructive partnerships with VGTx stakeholders, because only interdisciplinary scientific, industry, and game community partnerships will strengthen our capacity to engage in VGTx research.

New research frontiers require careful thought to the ethical, legal, and social implications of research on VGTx and the communities that play them. Protecting the privacy of players as research participants is an unclear landscape, with data privacy standards differing between game industry and research communities (49). Transparent descriptions of what type of play data will be used, how it will be used for research purposes, and the limits of data sharing will be vital to ensure participants’ privacy. Close collaboration will help ensure appropriate attention is given to guaranteeing the highest standards of research ethics, and that health research is not exploited for (or driven by) commercial gain. In addition, the potential adverse effects of any public health intervention should be considered. Clinical trials of VGTx will need to monitor and report all adverse effects. Finally, ways to address the conflicts of interest that might arise from academics working with industry partners (as is standard in areas such as pharmaceutical research) will have to be developed, including financial and non-financial conflicts of interest such as involvement of researchers with the games or the gaming communities being studied. Registration of clinical trials and hypothesis-testing in non-clinical studies is one way to enhance transparency and ensure reporting of outcomes (50).

A more nuanced and deeper understanding of the positive health and public health consequences of gaming as a major global pastime is long overdue. An industry that has experienced an approximately 8% compound annual growth rate since 2015 is, if anything, going to continue to penetrate further into contemporary society with continued innovation and novel digital gameplay (51, 52). It is contingent on the interdisciplinary research community to ramp up efforts to better understand which social and contextual elements of games and game communities can be harnessed as genuine health interventions, and which physical and mental health outcomes can be evaluated longitudinally on a population scale in a valid way.

## Conclusion: Make Way for Chocolate-Covered Strawberries

Our team has been exploring the clinical and curative applications of this oft-maligned space and recognizes the need for more evidence to support the widespread use of these innovations. To conclude, we present a call for strategic investment into a research agenda that includes:

- Developing standardized terminology and reporting to describe the complex aspects of commercial video games and gaming communities as exposures and interventions.
- Designing standardized protocols and best practice methodologies to evaluate the effects of therapeutic uses of games and gaming communities on clinical and public health outcomes.
- Investigating interdisciplinary approaches to capture and integrate rapidly changing technologies, game culture and communities, and games themselves.
- Establishing best practices and ethical protocols for rules of engagement with the game industry as partners, including standards for player privacy and management of conflicts of interest.
- Investigating best practices for identifying which games work for which health condition (and which population), including potential mechanisms for effects, economic analyses, and ongoing monitoring and evaluation of intervention effectiveness.
- Learning to model the complex, temporally dense data that games can provide.

Digital gaming today is a far cry from its 8-bit predecessors of the 1980s. Internet connectivity has fostered hyperconnected global communities of gamers and distributed social networks through play, the consequences and implications of which are poorly understood. This research agenda will require innovative public/private collaborations between academia, gaming communities, and private sector developers. Furthermore, research investments will need to be made to fill these important knowledge and research gaps.

As Carl Jung said, “The creation of something new is not accomplished by the intellect but by the play instinct” (53). We might be a decade or more away from important breakthroughs that leverage video gaming to heal people and populations. In comparison to games for health, which have been described as “chocolate-coated broccoli” (54), VGTx might be “chocolate-coated strawberries” designed to attract, thrill, and retain players, who reap inadvertent or intentional benefits of their gameplay. Learning how to harness these play possibilities for clinical and public health good will first require us in the public health and clinical research community to think outside the proverbial [X]box.

## Author Contributions

All authors contributed equally to the conception, writing and revision of the paper, approved the final version, and agreed to be accountable for all aspects of the work.

## Conflict of Interest Statement

The authors declare that the research was conducted in the absence of any commercial or financial relationships that could be construed as a potential conflict of interest. The reviewer SO and handling Editor declared their shared affiliation.
